# Selective block of adenosine A_2A_ receptors prevents ischaemic‐like effects induced by oxygen and glucose deprivation in rat medium spiny neurons

**DOI:** 10.1111/bph.15922

**Published:** 2022-07-27

**Authors:** Elisabetta Coppi, Alasdair J. Gibb

**Affiliations:** ^1^ Department of Neuroscience, Psychology, Drug Research and Child Health University of Florence Florence Italy; ^2^ Department of Neuroscience, Physiology and Pharmacology University College London London UK

**Keywords:** adenosine A2A receptor, anoxic depolarization, brain ischaemia, K+ currents, mediium spiny neurons, spontaneous and miniature excitatory postsynaptic currents

## Abstract

**Background and Purpose:**

Ischaemia is known to cause massive neuronal depolarization, termed anoxic depolarization (AD), due to energy failure and loss of membrane ion gradients. The neuromodulator adenosine accumulates extracellularly during ischaemia and activates four metabotropic receptors: A_1_, A_2A_, A_2B_ and A_3_. Striatal medium spiny neurons (MSNs) express high levels of A_2A_ receptors and are particularly vulnerable to ischaemic insults. A_2A_ Receptor blockade reduces acute striatal post‐ischaemic damage but the cellular mechanisms involved are still unknown.

**Experimental Approach:**

We performed patch‐clamp recordings of MSNs in rat striatal slices subjected to oxygen and glucose deprivation (OGD) to investigate the effects of A_2A_ receptor ligands or ion channel blockers on AD and OGD‐induced ionic imbalance, measured as a positive shift in E_rev_ of ramp currents.

**Key Results:**

Our data indicate that the A_2A_ receptor antagonist SCH58261 (10 μM) significantly attenuated ionic imbalance and AD appearance in MSNs exposed to OGD. The K^+^ channel blocker Ba^2+^ (2 mM) or the Na^+^ channel blocker tetrodotoxin (1 μM) exacerbated and attenuated, respectively, OGD‐induced changes.

Spontaneous excitatory post‐synaptic current (sEPSC) analysis in MSNs revealed that the A_2A_ receptor agonist CGS21680 (1 μM) prevented OGD‐induced decrease of sEPSCs within the first 5 min of the insult, an effect shared by the K^+^ channel blocker Ba^2+^, indicating facilitated glutamate release.

**Conclusion and Implications:**

Adenosine, released during striatal OGD, activates A_2A_ receptors that may exacerbate OGD‐induced damage through K^+^ channel inhibition. Our results could help to develop A_2A_ receptor‐selective therapeutic tools for the treatment of brain ischaemia.

## Graphical abstract

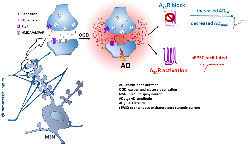

**Abbreviations**
AD: anoxic depolarization
APs: action potentials
mEPSCs: miniature excitatory post‐synaptic currents
MSN: medium spiny neuron
OGD: Oxygen and glucose deprivation
sEPSCs: spontaneous excitatory post‐synaptic currents
tMCAo: transient middle cerebral artery occlusion
tPA: tissue plasminogen activator

**What is already known**

- Adenosine A1, A2 and A3 receptor subtypes affect neuroprotective signalling during ischaemic brain damage.

**What does this study add**

- This study improves understanding of A2A receptor mechanisms during anoxic depolarisation in rat striatal neurons.

**What is the clinical significance**

- Results suggest A_2A_ receptor blockade may be protective by delaying and reducing anoxic depolarisation.

## 1 INTRODUCTION

Stroke is a major cause of brain damage and permanent disability worldwide but current treatments have limited therapeutic success. Much of post‐ischaemia neuronal death is triggered by exaggerated glutamate release (Choi & Rothman, 1990) resulting from a fall in cytoplasmic ATP concentration which causes a loss of plasma membrane Na^+^/K^+^‐ATPase activity and transmembrane ion gradients with consequent neuronal and glial depolarization. This condition induces an abrupt increase in extracellular K^+^ concentration ([K^+^]_o_) (Erecinska & Silver, 1994; Hansen, 1985) causing a massive release of neurotransmitters in the injured brain area and in particular an exaggerated glutamate release, partly due to vesicular release and mostly due to the reversal of glutamate transporters (Madl & Burgesser, 1993; Rossi et al., 2000; Szatkowski et al., 1990). Once released, extracellular glutamate activates ionotropic AMPA receptors as well as NMDA receptors that contribute to exaggerated depolarization and Ca^2+^ influx ultimately leading to anoxic depolarization (AD) and neuronal death (Obeidat & Andrew, 1998).

Whether an AD occurs during transient ischaemia and its time of occurrence are key factors influencing neurological deficits and the extent of the resulting brain damage. Adenosine signalling is a ubiquitous mechanism by which neurons and glial cells communicate with each other. Extracellular adenosine levels in the brain are generally in the nM range, but under pathological conditions such as brain ischaemia, adenosine is released in huge amounts (Pedata et al., 2016) and activates all subtypes of adenosine receptors, namely A_1_ , A_2A_ , A_2B_ and A_3_ receptor subtypes (Fredholm et al., 2011).

The increase in extracellular adenosine during hypoxia/ischaemia is thought to play a neuroprotective role through the activation of A_1_ receptors (Stone, 2005), which are highly expressed in the brain (Dunwiddie & Diao, 1994). The other G_i_‐coupled adenosine receptor subtype, the A_3_ receptor, proved to exert either protective (Pugliese et al., 2007) or detrimental effects (Pugliese et al., 2006; von Lubitz et al., 1999) during brain ischaemia, depending on the experimental model used or timing of A_3_ receptor ligand application. Interestingly, selective block of G_s_‐coupled A_2B_ receptors during and in vitro ischaemic‐like insult, that is, oxygen and glucose deprivation (OGD), is protective in acute hippocampal slices because it causes a delay in AD appearance and a reduction in CA1 astrogliosis, cytochrome C release and neuronal loss (Fusco et al., 2018), effects possibly mediated by inhibition of glutamate release (Fusco et al., 2019; Gonçalves et al., 2015). On the other hand, in in vivo models of transient middle cerebral artery occlusion (tMCAo), A_2B_ receptor agonists reduce brain damage, neuroinflammation and neurological deficit in rats (Dettori et al., 2021) possibly by inhibiting tissue plasminogen activator (tPA) and protecting cerebrovascular integrity (Li et al., 2017).

The other G_s_‐coupled adenosine receptor, the A_2A_ receptor, is known to be a crucial mediator of ischaemic injury in the hippocampus (Maraula et al., 2013) and striatum (Pedata et al., 2016). Its block by the A_2A_ receptor antagonist SCH58261 during acute ischaemic injury, or during the post‐ischaemic phase (i.e. within 24 h after tMCAo), proved protective by reducing neurotransmitter outflow, histological damage and neurological impairment (Melani et al., 2003). Furthermore, SCH58261 also prevented oxidative stress and neuronal injury after acute hypoxic insults in the striatum of new‐born piglets (Ortega‐Gutierrez et al., 2020). However, when the A_2A_ receptor antagonist was administered more than 7 days after the insult, the protection from neurological deficit was lost (Melani et al., 2015). Indeed, the administration of the selective A_2A_ receptor agonist CGS28561 at later phases after the insult (i.e. 7 days after tMCAo) protected the post‐ischaemic brain tissue by reducing peripheral immune cell infiltration (Melani et al., 2014), in line with the well‐known anti‐inflammatory role of A_2A_ receptors in peripheral cells (Antonioli et al., 2013). Taken together, the above data demonstrate that ischaemia‐induced early excitotoxicity can be relieved by A_2A_ receptor antagonists whereas, at later times after the insult, secondary neuroinflammation requires A_2A_ receptor agonist‐mediated neuroprotection.

Of note, no data have been reported to date about the effect of A_2A_ receptor ligands in MSNs during an OGD insult. In the present study, we performed patch‐clamp recordings in MSNs from acute striatal slices to elucidate the mechanisms by which an ischaemic‐like insult, obtained in vitro by OGD, causes functional impairment in these cells and to study the involvement of A_2A_ receptors in these events.

## 2 METHODS

### 2.1 Solutions

Slicing solution was (in mM): sucrose, 206; KCl, 2.5; CaCl_2_ 1, MgCl_2_ 4; NaH_2_PO_4_ 1.25, NaHCO_3_ 25, kynurenic acid 0.1, glucose 25, pH 7.4 when saturated with 95% O_2_ and 5% CO_2_. Recording solution was (in mM): NaCl 125, KCl 2.5, CaCl_2_ 1, MgCl_2_ 1, NaH_2_PO_4_ 1.25, NaHCO_3_ 25, glucose 25, pH 7.4 when saturated with 95% O_2_ and 5% CO_2_. Slicing and recording solutions were gassed continuously with a mixture of O_2_ (95%) and CO_2_ (5%). OGD solution was obtained by replacing glucose with equimolar sucrose in the recording solution and by bubbling with 95% N_2_ and 5% CO_2_. Pipette solution was (in mM): K‐gluconate 130, NaCl 4.8, MgCl_2_ 2, CaCl_2_ 1, EGTA 0.2, HEPES 10, adjusted to pH 7.4, with KOH. Pipette solution was stored at −20°C in 1 ml aliquots. Before starting the experiment, Na_2_ATP 2 mM and NaGTP 0.3 mM were added daily to each aliquot of pipette solution stored on ice during use.

### 2.2 Brain slice preparation

Striatal slices were prepared as described elsewhere (Coppi et al., 2012). Briefly, male Sprague–Dawley rats were group‐housed in a temperature (22 ± 1°C) and humidity‐controlled environment with 12 h light/dark illumination cycle and with food and water *ad libitum*. Animal care followed the guidelines of the UK Home Office and the UCL Institutional Animal Care and Use Committee. At postnatal day 28 (P28) animals were deeply anaesthetized with isofluorane and decapitated in accordance with the UK Animals (Scientific Procedures) Act 1986 and Local Ethical Committee approval. Animal studies were in compliance with the ARRIVE guidelines (Percie du Sert et al., 2020) and with the recommendations made by the British Journal of Pharmacology (Lilley et al., 2020). Every effort was made to minimize animal suffering and the number of animals used. The brain was removed from the skull and submerged in ice‐cold oxygenated slicing solution. Coronal brain slices (300 μm thick) were prepared using a vibrating microslicer (Leica VT1000, Wetzlar, Germany) and incubated at room temperature (20–24°C) in oxygenated recording solution for 1–6 h before use. A single slice was then transferred into a 0.3 ml incubation chamber continuously superfused (2 ml·min^−1^) with oxygenated recording solution by a six‐way, gravity‐fed system. When indicated, A_2A_ receptor ligands or ion channel blockers were added to the recording solution and perfused for at least 10 min before OGD. In particular, the Na^+^ channel blocker tetrodotoxin (TTX, 1 μM), the K^+^ channel blocker Ba^2+^ (2 mM), the A_2A_ receptor agonist CGS21680 (1 μM) or the A_2A_ receptor antagonist SCH58261 (10 μM) were used in the present research. CGS21680 and SCH58261 concentrations were chosen (using the Gaddum equation) to give receptor occupancy ~90% in control and to reduce adenosine receptor occupancy during OGD to less than 10% in presence of SCH58216 assuming extracellular adenosine concentrations of 100 nM and 20 μM in control and during OGD respectively (Pedata et al., 2016).

### 2.3 Data and statistical analysis

The present research was performed on n = 21 control cells recorded from 21 slices prepared from 16 animals, n = 12 cells recorded from 12 SCH58261‐treated slices prepared from five animals, n = 11 cells recorded from 11 CGS21680‐treated slices prepared from five animals, n = 10 cells recorded from 10 Ba^2+^‐treated slices prepared from 5 animals and n = 10 cells recorded from 10 TTX‐treated slices prepared from 5 animals. Each slice recording was made independently and recordings were grouped per animal for statistical analysis. Sample size estimation and power analysis was performed using G*Power (Version 3.1.9.6 – available from https://gpower.software.informer.com/3.1/). Randomisation and blinding of slice treatments was not done due to the nature of the primary experimental manipulation (e.g. change to solution with oxygen and glucose deprivation). All statistical comparisons between groups were made for n = 5 or more rats per groups as detailed above.

Shapiro–Wilk normality test was performed to check data distribution. As most of the data tested negative (i.e. not normally distributed), statistical analysis was made uniformly with non‐parametric tests, unless otherwise stated. Averaged data are reported as median ± 95% confidence interval (CI). Wilcoxon or Dunn's multiple comparison non‐parametric tests were performed, as appropriate, in order to determine statistical significance (*P* < 0.05). Data were analysed using ‘GraphPad Prism’ (San Diego, CA, USA) software. The data and statistical analysis comply with the recommendations of the *British Journal of Pharmacology* on experimental design and analysis in pharmacology (Curtis et al., 2018). Technical replicates were used to ensure the reliability of single values. No exclusion criteria were applied to any of the experimental data.

### 2.4 Whole‐cell recordings

Patch pipettes were pulled from thick‐walled borosilicate glass capillaries (GC150F‐7.5, Harvard Apparatus, Holliston, MA, USA) to final resistance of 4–7 MΩ. Membrane voltages given in the results are corrected for a calculated liquid junction potential of −8 mV. To reduce the voltage error due to the series resistance (Rs), 75%–80% of the Rs was compensated before starting recordings. The cell bodies of individual neurons in brain slices were visualized under Nomarski differential interference contrast optics. Medium spiny neurons (MSNs) comprise about 90% of striatal neurons (Rymar et al., 2004) and are characterized by a resting membrane potential (RMP) around −80 mV, a small cell diameter (~20 μm) and a membrane resistance (R_m_) of about 300 MΩ (Cao et al., 2018). The present research was performed on n = 64 cells identified as striatal MSNs on the basis of their location, size, morphology and electrophysiological properties: cells had a mean RMP of −80.2 ± 0.7 mV, a cell capacitance (C_m_) of 22.3 ± 1.6 pF and a R_m_ of 185.6 ± 17.6 MΩ.

### 2.5 Voltage‐clamp experiments

Whole‐cell currents were recorded as previously described (Coppi et al., 2012). Briefly, recordings were made using an Axopatch 200B amplifier (Axon Instruments, Foster City, CA, USA), filtered at 1 kHz (8‐pole Bessel) and digitized at 10 kHz using an analogue‐to‐digital converter (CED micro 1401, Cambridge Electronic Design, UK). Cells were voltage clamped at −60 mV and the holding current (I_h_) continuously monitored throughout the experiment. Four successive voltage ramps (from −118 to −63 mV, 1 s duration, 2 s inter‐episode interval) were applied to the cell every 1 min and recorded using the program WinEDR (available from Strathclyde Electrophysiology Software, Glasgow, UK, at https://spider.science.strath.ac.uk/sipbs/software_ses.htm). All ramp traces shown in the figures are the average of four consecutive ramp episodes collected at 2 s inter‐episode interval. Spontaneous excitatory post‐synaptic currents (sEPSCs) in MSNs were recorded at −60 mV throughout the experiment and detected by using the ‘template’ function in the program WinEDR. When TTX was present in the bath solution, spontaneous AP‐independent miniature excitatory post‐synaptic currents (mEPSCs) were recorded by the same method. Traces were digitally filtered (low pass 540 Hz) for analysis.

### 2.6 Oxygen and glucose deprivation (OGD)

Experiments were performed as described (Fusco et al., 2018). Briefly, after a stable baseline (less than ~10% variation) of I_h_ and ramp currents was acquired for at least 5 min, OGD was applied by switching to a glucose‐free and oxygen‐deprived (i.e. continuously bubbled with a mixture of 95% N_2_ and 5% CO_2_) recording solution. OGD was continued until 1 min after the appearance of the AD peak, measured by increased I_h_. After the AD was completed (i.e. when I_h_ reached a negative steady‐state level), slices were reperfused with standard, glucose‐containing and oxygenated, recording solution. Changes in membrane ionic balance during OGD were monitored by quantifying the ‘zero current potential’ (E_rev_) of ramp currents before and during the insult. E_rev_ was measured by interpolating each averaged ramp trace with a polynomial equation and solving for ‘y = 0’. Changes in R_m_ during OGD were quantified by isolating averaged ramp traces between −90 and −70 mV and fitting a straight line to those data points. The value of the slope corresponds to the inverse of R_m_.

All pre‐OGD values were measured 2 min before OGD start. AD latency was measured as the time between OGD start and the time at which I_h_ increased by more than 20% of pre‐OGD level. The ‘AD peak time’ was measured as the time between OGD start and the time at which I_h_ reached its maximal negative peak. The latency between I_h_ change >20% and AD peak was measured as the difference between AD latency and ‘AD peak time’. AD peak amplitude was measured as the difference between pre‐OGD I_h_ value and the maximum negative peak recorded during OGD. The latency for R_m_ decrease during OGD was measured as the time between OGD start and the time at which R_m_ changed more than 20% from pre‐OGD value. Changes in E_rev_ during OGD were measured by subtracting the pre‐OGD value from that measured between 15 and 18 min OGD. Assuming this is dominated by changes in [K^+^]_o_, the predicted [K^+^]_o_ before or during OGD, E_rev_ was extrapolated from Nernst's equation:

$$E_{\mathrm{rev}}=\mathrm{RT}/\mathrm{zF}\;\ln\left({\left[K^{+}\right]}_{o}/{\left[K^{+}\right]}_{i}\right)$$

where *z* is the ion charge, *R* is the gas constant, *F* is the Faraday's constant and *T* is the temperature in °K, by substituting respective E_rev_ values measured as the ‘zero current potential’ of four averaged voltage ramps elicited 1 min before OGD or during AD peak. The latency to E_rev_ inflection point was measured as the time between OGD start and the time of inflection point (the time at which the straight line interpolating data points in the pre‐OGD phase crosses the straight line interpolating data points during I_h_ increase >20% until the end of OGD).

### 2.7 Drugs and chemicals

NaCl, NaOH, NaH_2_PO_4_, NaHCO3, KCl, BaCl_2_, CaCl_2_, MgCl_2_, sucrose, CsCl, NMDG and glucose were purchased from BDH Laboratory Supplies (Poole, England). HEPES, EGTA, ATP and GTP, were purchased from Sigma (St. Louis, MO). Tetrodotoxin (TTX) was purchased from Ascent Scientific (Bristol, UK) or Alomone Labs (Jerusalem, Israel). The concentrations of the A_2A_ receptor agonist CGS21680 (1 μM) and antagonist SCH58261 (10 μM) were chosen on the basis of ligand‐binding studies from Cunha et al. (1999) and from Zocchi et al. (1996), respectively.

### 2.8 Nomenclature of targets and ligands

Key protein targets and ligands in this article are hyperlinked to corresponding entries in the IUPHAR/BPS Guide to PHARMACOLOGY http://www.guidetopharmacology.org and are permanently archived in the Concise Guide to PHARMACOLOGY 2021/22 (Alexander, Christopoulos et al., 2021; Alexander, Fabbro et al., 2021; Alexander, Kelly et al., 2021; Alexander, Mathie et al., 2021).

## 3 RESULTS

### 3.1 OGD‐induced appearance of anoxic depolarization in rat striatal MSNs is delayed by the A_2A_ receptor antagonist SCH58261

**FIGURE 1:**
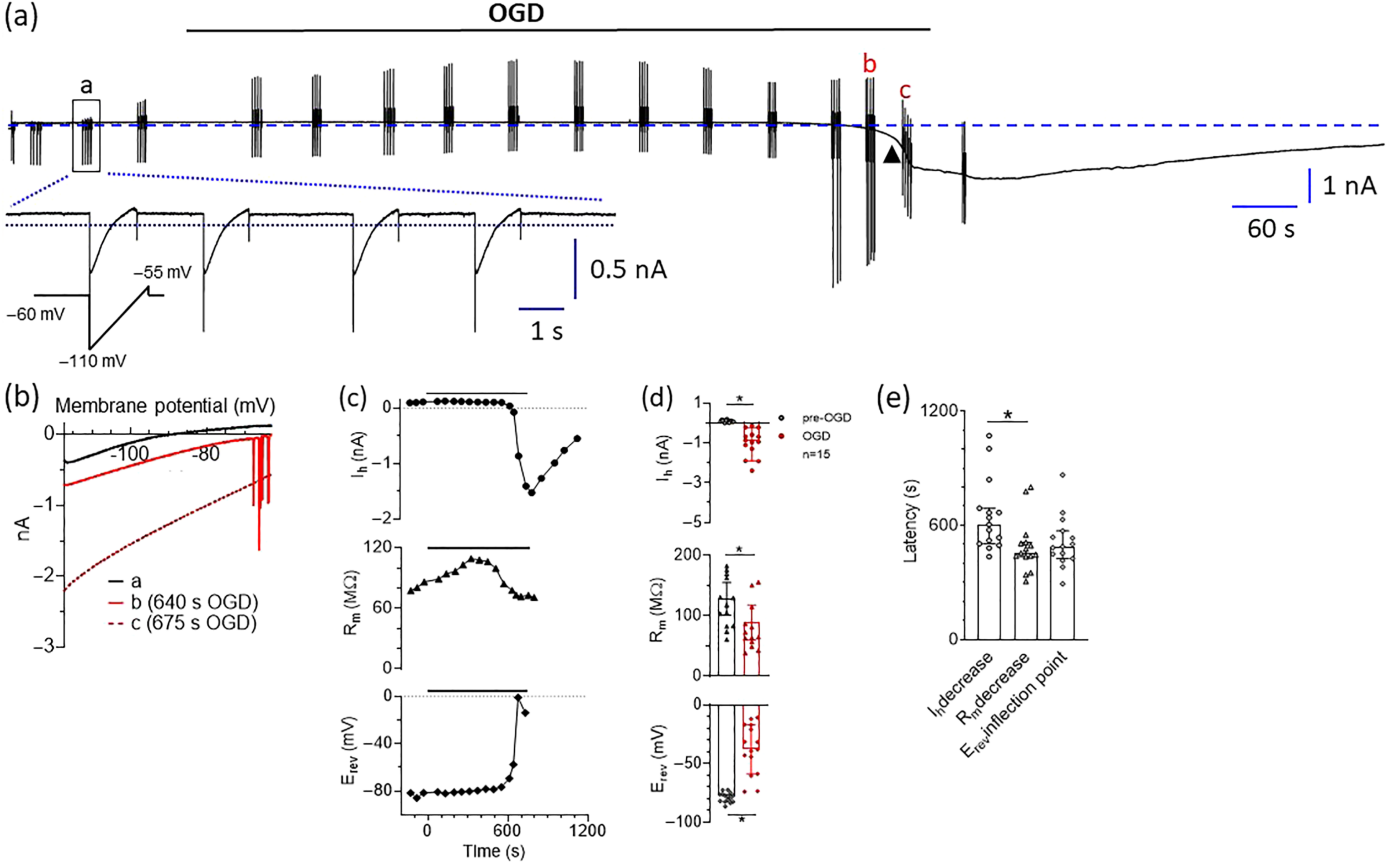
A prolonged oxygen and glucose deprivation (OGD) insult in striatal medium spiny neurons triggers a series of electrophysiological changes which ultimately lead to the appearance of anoxic depolarization (AD). **(a)** Representative whole‐cell patch‐clamp trace recorded at −60 mV from a medium spiny neuron (MSN) in a P28 rat striatal slice subjected to OGD conditions. OGD was applied until the sudden increase (arrowhead) in holding current (I_h_) reached a steady state level (710 s in this representative cell). *Lower inset*: Expanded time scale of the four voltage ramp traces (from −113 to −63 mV, duration 1 s; 2 s inter‐episode interval) from box ‘a’ is shown as an example of the measurement of the membrane resistance (R_m_) and the current reversal potential (E_rev_). A trial consisting of four ramps was applied in each recorded cell every 60 s. **(b)** Current–voltage (I‐V) relationship of ramp traces recorded in the same cell at different time points: before OGD (trial at a in **(a)**), during OGD but immediately before anoxic depolarization (AD: trial at b in **(a)**) and during AD (trial at c in **(a)**). Each ramp is the average of four individual voltage traces within each trial examined. **(c)** Time course of changes in I_h_ (*upper panel*), R_m_ (*middle panel*) and E_rev_ (*lower panel*) in the same cell. **(d)** Pooled data (median ± 95% confidence interval: CI) of I_h_ (*upper panel*), R_m_ (*middle panel*) and E_rev_ (*lower panel*) measured in 15 cells before (pre‐OGD: last 2 min before OGD) or during OGD (last 2 min of OGD). *P < 0.05; Wilcoxon test. **(e)** Pooled data of the latency (measured from OGD start) to reach I_h_ increase >20% (circles), R_m_ decrease >20% (triangles) or E_rev_ inflection point (diamonds) in 21 cells recorded. *P < 0.05; Dunn's multiple comparisons test

We performed patch‐clamp recordings from rat striatal MSNs in order to detect whether the electrophysiological changes induced by an OGD insult in these cells are modified by A_2A_ receptor selective block. The amplitude of I_h_ was measured before and during OGD by clamping MSNs to −60 mV. Furthermore, in order to monitor estimated changes in [K^+^]_o_ and/or R_m_, we applied voltage ramp protocols before, during and after the insult (Figure 1a; lower inset).

As shown in Figure 1a, a prolonged OGD insult caused an abrupt increase in I_h_ at −60 mV. This event is commonly called anoxic depolarization (AD) and is primarily due to a loss of electrochemical gradients and consequent neuronal depolarization, exacerbated by extracellular glutamate accumulation (Allen et al., 2004; Rossi et al., 2000). Immediately before AD, a series of events are observed which can be recapitulated as follows: (1) a ‘slow phase’ of electrophysiological changes consisting of I_h_ increase (see Figure 1a between ‘a’ and ‘b’) and a gradual positive shift in the ‘0 current’ potential of the voltage ramp likely resulting from loss of K^+^ from neurons in the slice and opening of neurotransmitter‐gated ion channels (Figure 1b; see differences between trace ‘a’ and trace ‘b’); (2) sporadic downward deflections observed during the voltage ramp at potentials around −65/−55 (Figure 1b; trace b) representing action potentials (APs) detected as inward action currents in voltage‐clamp mode; (3) a massive increase in inward ramp currents simultaneous to a marked shift in the ramp ‘zero current potential’ (Figure 1b; trace c) and, lastly, (4) a drop in I_h_ at −60 mV (arrowhead in Figure 1a) corresponding to the AD. Concerning point 2, repetitive AP firing was observed in 67% of MSNs (10 out of 15 cells recorded). These currents were never observed when OGD was performed in the presence of TTX (see below). The increase in ramp currents (point 3) is consistent with a reduction in R_m_ during OGD whereas the positive shift in E_rev_ is mainly indicative of changes in the reversal potential for K^+^ ions due to extracellular K^+^ accumulation during anoxia (Hansen, 1985). Figure 1c shows the time course of changes in I_h_, R_m_ and E_rev_ in a representative MSN subjected to OGD. These changes were statistically significant when measured in 15 control OGD neurons (Figure 1d) and are in line with previous results in the literature from hippocampal CA1 neurons (Rossi et al., 2000). Of note, the calculated [K^+^]_o_ at AD peak, measured by extrapolation from respective E_rev_ values by the Nernst equation, was 30.0 ± 1.2 mM, consistent with previous data from the hippocampus reported in the literature (Hansen, 1985).

Furthermore, the latency to R_m_ decrease (>20% change from pre‐OGD level) measured in 15 control OGD experiments was 7.6 min (454 s: central bar in Figure 1e), a value significantly smaller than that observed for I_h_ increase, that is, 10 min (604 s: left bar in Figure 1e). In contrast, the latency to E_rev_ shift (inflection point in E_rev_) was 8.1 min (488.8 s: right bar in Figure 1e), a value not significantly different from the latency to R_m_ nor I_h_ changes.

**FIGURE 2:**
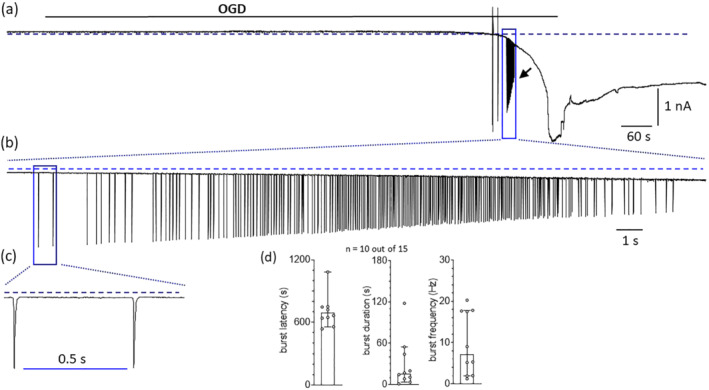
In a subset of striatal medium spiny neurons, OGD induced a burst of repetitive action potentials during anoxic depolarization (AD). **(a)** Original patch‐clamp current trace recorded in a −60 mV clamped medium spiny neuron (MSN) from a P28 rat striatal slice where an oxygen and glucose deprivation (OGD) insult was applied. In this particular cell no voltage ramps were elicited. OGD was prolonged until the increase of holding current (I_h_) reached a steady state level (980 s in this particular MSN). Arrow indicates the burst of spontaneous action potentials (APs) that accompanies the AD phase. **(b, c)** Progressively expanded time scales of the same event. **(d)** Pooled data (median ± 95% confidence interval: CI) of burst latency (*left panel*), duration (*central panel*) or frequency (*right panel*) in 10 out of 15 slices in which the spontaneous burst was recorded

As mentioned above (point 2), in some cases (10 out of 15 MSNs recorded, 67%), a spontaneous burst of APs (arrow in Figure 2a and expanded time scales in Figure 2b,c) was observed during AD. This phenomenon has been previously described by others during hypoxia (Guatteo et al., 1998; Karunasinghe & Lipski, 2013). When observed, the latency to spontaneous AP burst was 12.6 min (692.5 s) from OGD start (Figure 2d; left panel), lasted for 15.7 s (Figure 2b; central panel) and had an averaged frequency of 7.2 Hz (Figure 2d; right panel).

**FIGURE 3:**
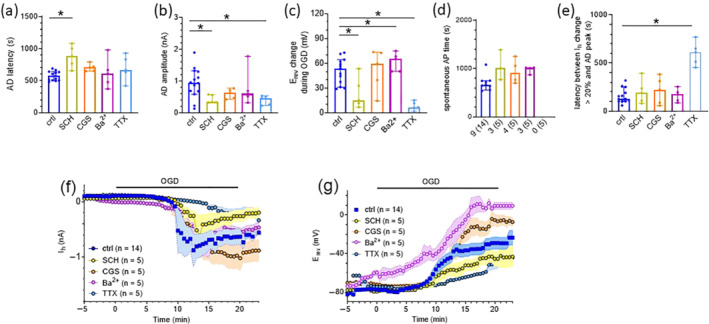
The A_2A_ receptor antagonist SCH58261 delayed the appearance of anoxic depolarization (AD) induced by OGD in rat striatal medium spiny neurons. **(a–e)**. Pooled data (median ± 95% confidence interval: CI) of AD latency (measured as the time needed for I_h_ to change more that 20% from OGD start: **(a)**), AD amplitude (measured as the difference between pre‐OGD I_h_ value and the AD peak: **(b)**), E_rev_ changes during OGD (measured as the difference between pre‐OGD E_rev_ value and the value reached during the AD peak: **(c)**), the latency to initiate the spontaneous action potential (AP) burst (when detected) **(d)** and the latency between I_h_ change (more than 20%) and AD peak **(e)** in striatal medium spiny neurons (MSNs) in different experimental conditions: untreated OGD slices (ctrl; blue circles) or slices subjected to OGD in the presence of different pharmacological treatments: the A_2A_ receptor antagonist SCH58261 (SCH, 10 μM; yellow circles); the A_2A_ receptor agonist CGS21680 (CGS, 1 μM; orange circles); the K^+^ channel blocker Ba^2+^ (2 mM; purple circles) or Na^+^ channel blocker tetrodotoxin (TTX, 1 μM; green circles). Each compound was added at least 10 min before OGD and throughout the insult. Note that the proportion of MSNs presenting the burst of APs during OGD is reported below each column in panel **(d)**. **(f, g)** Averaged time courses of I_h_ (**f**) or E_rev_ (**g**) before or during OGD performed in different experimental groups. *P < 0.05; Dunn's multiple comparisons test

We then investigated the effect of selective A_2A_ receptor ligands on the OGD‐induced changes in electrophysiological parameters described above. When OGD was performed in the presence of the A_2A_ receptor antagonist SCH58261 (10 μM), AD appearance was significantly delayed (Figure 3a) and E_rev_ depolarization was significantly smaller (Figure 3c). Of note, only 3 out of 5 (60%) experiments performed with SCH58261 presented a spontaneous AP burst during AD. On the other hand, when OGD was performed in the presence of the A_2A_ receptor agonist CGS21680 (1 μM), neither AD latency, amplitude nor E_rev_ changes were different from control (Figure 3a–c). Of note, 4 out of 5 (80%) experiments performed in CGS21680 presented spontaneous AP bursts during AD. However, the latency of this phenomenon was similar to what was observed in control slices (Figure 3d).

We then tested the involvement of voltage‐gated K^+^ or Na^+^ channels in OGD‐induced electrophysiological changes by performing OGD in the presence of K^+^ or Na^+^ channel blockers: Ba^2+^ (2 mM) or TTX (1 μM), respectively. As shown in Figure 3a, neither of these compounds affected AD latency. However, AD amplitude and E_rev_ changes during OGD were significantly attenuated in the presence of TTX (Figure 3b,c) and, in addition, the latency between AD appearance (>20% increase in I_h_) and AD maximum I_h_ (lowest value of I_h_ reached) was significantly increased (Figure 3e), indicating that TTX decelerates the rate at which AD deflection occurs before the I_h_ drop. As expected, none of the MSNs investigated presented spontaneous bursting activity in the presence of TTX (Figure 3c), thus confirming that this phenomenon depends on voltage‐gated Na^+^ channel‐dependent AP firing.

As summarized in Figure 3f, the selective block of A_2A_ receptors delayed AD appearance and reduced its amplitude, whereas their activation exacerbated AD. Similarly, E_rev_ depolarization was significantly decreased by the A_2A_ receptor blocker SCH58261 and enhanced by the A_2A_ receptor agonist CGS21680. Furthermore, K^+^ channel block exacerbated, whereas Na^+^ channel block mitigated, OGD‐induced changes in I_h_ and E_rev_.

**FIGURE 4:**
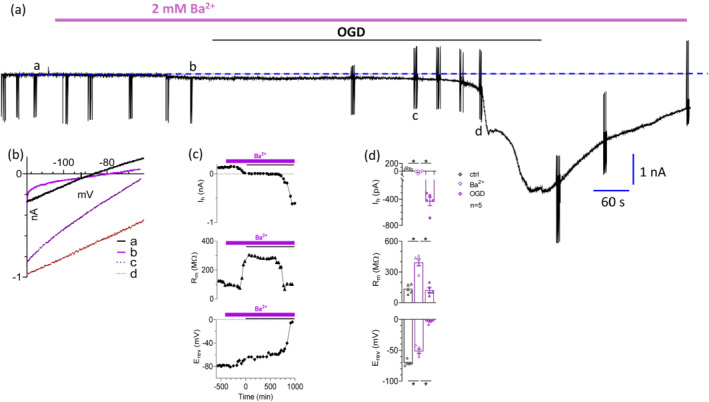
Ba^2+^‐induced electrophysiological changes before or during oxygen and glucose deprivation (OGD) in striatal medium spiny neurons. (a) Original patch‐clamp current trace recorded in a −60 mV clamped medium spiny neuron (MSN) from a p28 rat striatal slice where an OGD insult was applied in the presence of extracellular Ba^2+^ (2 mM). OGD was prolonged until the increase in holding current (I_h_) reached a steady state level (760 s in this particular MSN). Downward deflections are ramp protocols (from −113 to −63 mV in 1 s, 2 s inter‐episode interval) evoked to measure the membrane current reversal potential (E_rev_) and membrane resistance (R_m_) at different time points. A trial consisting of four consecutive ramp episodes was repeated every 60 s. (b) Current‐to‐voltage (I‐V) relationship of ramp traces recorded in the same cell at different time points: before the application of Ba^2+^ (trial at a in (a)), after 5 min of Ba^2+^ application and before OGD (trial at b in (a)), during OGD but immediately before anoxic depolarization (AD: trial at c in (a)) and during AD (trial in c). Each ramp is the average of four individual voltage traces within each trial examined. (c) Time courses of I_h_ (*upper panel*), R_m_ (*middle panel*) and E_rev_ (*lower panel*) recorded in the same representative cell. (d) Pooled data (mean ± SEM) of I_h_ (*upper panel*), R_m_ (*middle panel*) and E_rev_ (*lower panel*) changes measured in control conditions (ctrl: grey symbols), in Ba^2+^ (open purple symbols: last 2 min before OGD in Ba^2+^) or during OGD in Ba^2+^ (filled purple symbols: last 2 min of OGD in Ba^2+^). *P < 0.05, paired Student's *t*‐test

The effects of the K^+^ channel blocker Ba^2+^ on OGD‐induced changes are reported in detail in Figure 4. During the pre‐OGD phase, Ba^2+^ (2 mM) caused a modest, but significant, increase in the I_h_ at −60 mV indicating, as expected, neuronal depolarization upon K^+^ channel block (Figure 4a). Indeed, I_h_ was usually measured as a positive value in MSNs in our experimental conditions, consistent with the fact that the resting membrane potential (RMP) of these cells is about −70 mV or below (Cao et al., 2018). After 5 min of Ba^2+^ application, I_h_ significantly decreased (Figure 4c,d; upper panels: from 62.7 ± 8.9 pA in control to −14.2 ± 9.3 pA in 2 mM Ba^2+^, n = 5). Furthermore, a significant increase in R_m_ (Figure 4c,d; middle panels) and a positive shift in E_rev_ (Figure 4c,d; lower panels: calculated [K^+^]_o_ in Ba^2+^ during the pre‐AD phase was 15.2 ± 1.1 mM) were measured in the pre‐OGD period, in line with K^+^ channel block. Then, when OGD was carried out in the presence of extracellular Ba^2+^, I_h_ change was similar to the control group: a slow increase in I_h_ (Figure 4c,d; lower panels) preceded I_h_ drop corresponding to the AD. Of note, the E_rev_ increase during OGD was markedly exacerbated in Ba^2+^‐treated slices, consistent with a more pronounced accumulation of extracellular K^+^ in these conditions. In fact, the estimated [K^+^]_o_ at AD peak when extracellular Ba^2+^ is present was 118.7 ± 3.4 mM, indicating an exacerbation of extracellular K^+^ overload during OGD.

Finally, it is note‐worthy that AD appearance during OGD is known to be temperature sensitive (Rosen & Morris, 1994). So, we continuously monitored the temperature of the recording chamber before, during and after OGD. Figure S1 shows that no significant differences were found in the bath temperature during OGD among different experimental groups (Figure S1).

### 3.2 OGD induced inhibition of spontaneous synaptic current frequency in MSNs is prevented by the A_2A_ receptor agonist CGS58261 and by K + channel block

**FIGURE 5:**
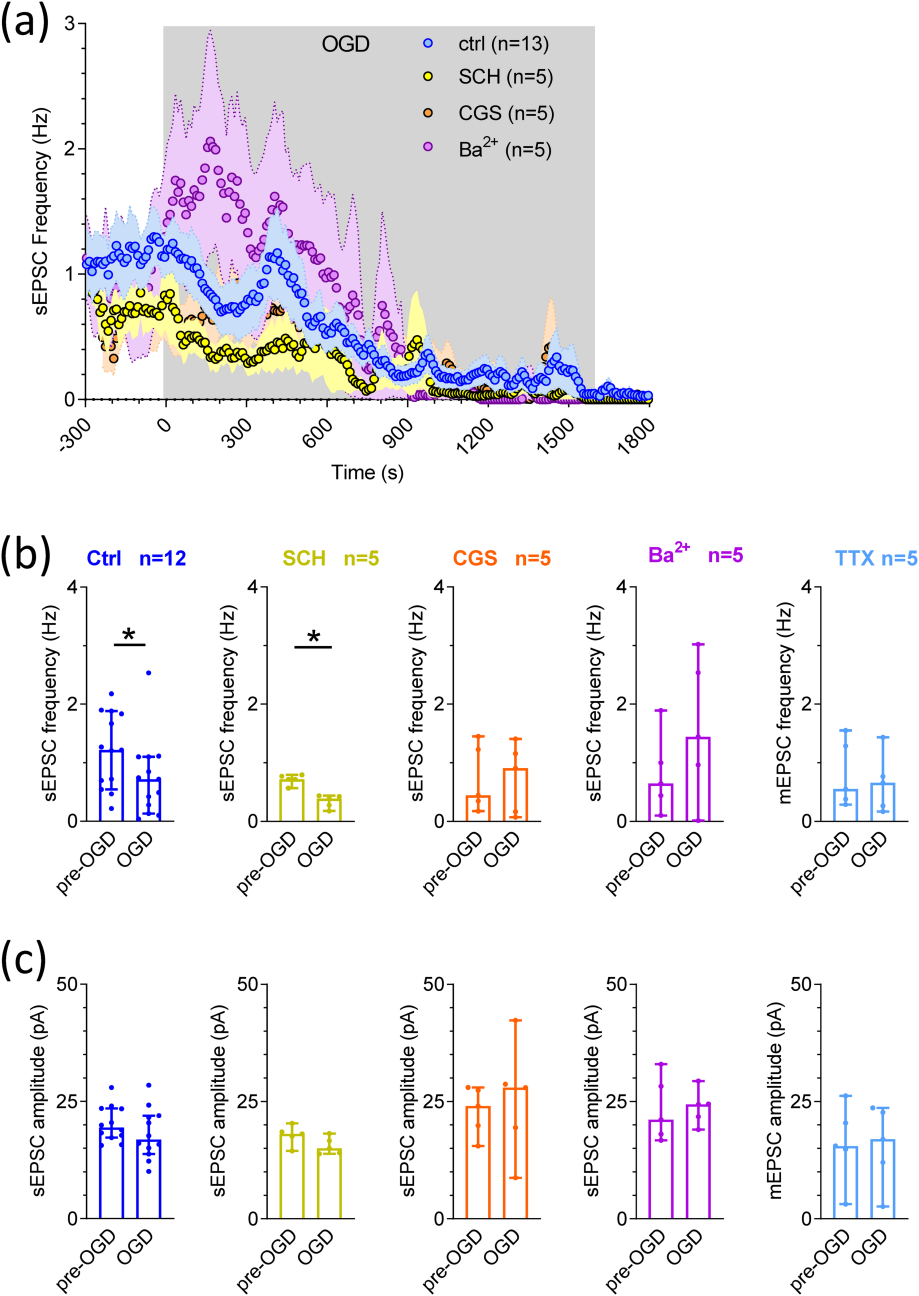
Oxygen and glucose deprivation (OGD) caused a decrease in the frequency of spontaneous, but not miniature, excitatory post‐synaptic currents in medium spiny neurons, an effect prevented by K^+^ channel block and by the selective stimulation of A_2A_ receptors. **(a)** Averaged time courses of spontaneous or miniature EPSCs (sEPSCs or mEPSCs, respectively) event frequency recorded in MSNs subjected to OGD in different experimental conditions: in control conditions (ctrl: blue circles; n = 12); in the presence of the A_2A_ receptor antagonist SCH58261 (SCH, 10 μM: yellow circles; n = 5); in the presence of the A_2A_ receptor agonist CGS21680 (CGS, 1 μM: orange circles; n = 5); in Ba^2+^ (2 mM: purple circles; n = 5) or in tetrodotoxin (TTX, 1 μM: green circles; n = 5). **(b, c)** Pooled data (median ± 95% confidence interval: CI) of event frequency (b) or amplitude (c) measured during the last 2 min before OGD (pre‐OGD) or between 3 and 5 min OGD (OGD) in different experimental groups. *P < 0.05; Wilcoxon test

In order to gain insight into the mechanisms by which A_2A_ receptors contribute to OGD‐induced neuronal damage, we measured the frequency and amplitude of spontaneous excitatory post‐synaptic currents (sEPSCs) as an index of overall striatal synaptic excitability before or during the ischaemic‐like insults carried out in the presence of drugs or in Ba^2+^ or in control conditions. As shown in Figure 5, following initiation of OGD there was a significant decrease in sEPSC frequency (Figure 5a,b; blue symbols) in the period between 3–5 min of OGD, indicating a decreased probability of glutamate release as the insult starts, before proceeding to a complete loss of synaptic transmission likely due to energy depletion. No changes were measured in sEPSC amplitude (Figure 5c; left panel). When OGD was carried out in the presence of the A_2A_ receptor antagonist SCH58261 (10 μM), the decrease in sEPSC frequency was preserved (Figure 5a,b; yellow symbols) but, at variance, it was completely prevented by the selective A_2A_ receptor agonist CGS21680 (1 μM: Figure 5a,b; orange symbols). This result indicates that selective A_2A_ receptor activation counteracts OGD‐induced inhibition of neurotransmitter release. A similar effect was observed when OGD was performed in the presence of extracellular Ba^2+^ (2 mM): OGD‐induced decrease in sEPSC frequency was absent during K^+^ channel block (Figure 5a,b; purple symbols). Of note, no significant changes were observed in the sEPSC amplitude before or during OGD in any of the experimental groups investigated (Figure 5c), suggesting no differences in the expression level of AMPARs on the post‐synaptic membrane.

Finally, when TTX (1 μM) was added to the extracellular solution, spontaneous mEPSCs (miniature excitatory post‐synaptic currents) were observed. Unlike sEPSCs which are AP‐dependent synaptic events, mEPSCs give a measure of spontaneous AP‐independent quantal neurotransmitter release (Wu et al., 2007). Our data demonstrate that OGD did not influence the frequency nor the amplitude of mEPSCs in rat MSNs suggesting no change in the post‐synaptic response to glutamate release (Figure 5a–c; green symbols; data not shown).

## 4 DISCUSSION

The present work demonstrates, for the first time, that the selective block of A_2A_ receptors in rat striatal slices protects MSNs from OGD‐mediated insults by delaying the appearance of AD.

Brain ischaemia is known to produce severe neurological impairments associated with neuroinflammation and neurodegeneration. Much of ischaemia‐induced neuronal death is triggered by exaggerated glutamate release (Choi & Rothman, 1990; Rossi et al., 2000; Tambasco et al., 2018). Extracellular K^+^ increase and massive neuronal depolarization are among the main triggers of glutamate‐mediated excitotoxicity and significantly contribute to metabolic imbalance and AD appearance (Blank & Kirshner, 1977). However, the failure of glutamate receptor antagonists to protect from stroke‐induced neuronal death (Ikonomidou & Turski, 2002) in the clinic has stimulated research towards new approaches for the treatment of brain ischaemia, including those based on avoiding uncontrolled neuronal depolarization upon energy failure. Encouraging results came from studies aimed to promote K^+^ channel opening after stroke. Early treatment with K^+^ channel openers, in particular those targeting ATP‐sensitive K^+^ channels, blocked the expression of a number of ischaemia‐induced immediate early genes and protected neuronal cells from degeneration (Heurteaux et al., 1993). Furthermore, the prototype M‐channel ‘opener’ retigabine reduced the cascade of deleterious events following traumatic brain injury, including hyperexcitability, ischaemia/hypoxia‐related metabolic stress and cell death (Vigil et al., 2020). These studies suggest that early reduction of neuronal excitability and energy demand via K^+^ current enhancement may provide an efficacious therapeutic intervention against post‐ischaemic brain damage.

Adenosine is an important neuromodulator in the CNS and is massively released during hypoxic/ischaemic conditions (Pedata et al., 2016). Interestingly, A_2A_ receptors, which are highly expressed in the striatum, specifically on dopamine D2 receptor‐containing MSNs (Le Moine et al., 1997), are associated with K^+^ channel inhibition in different cell types and, vice versa, A_2A_ receptor antagonists induce K^+^ channel opening in several experimental models (Coppi et al., 2013; Duffy et al., 2007; Saegusa et al., 2004). It could be argued that the mechanism by which A_2A_ receptor block in MSNs during OGD delays AD appearance is the enhancement of K^+^ currents and consequent reduction of cell excitability. This hypothesis is in line with the fact that, when OGD is carried out in the presence of the A_2A_ receptor antagonist SCH58261, E_rev_ shift is significantly reduced suggesting a less severe extracellular [K^+^] overload, and AD latency is delayed, indicating that striatal MSNs can endure a longer OGD period before undergoing irreversible energy failure. In contrast, the selective A_2A_ receptor agonist CGS21680, as well as the K^+^ channel blocker Ba^2+^, enhanced the E_rev_ shift and AD amplitude. Furthermore, while OGD decreased sEPSC frequency in the first phases of the insult, either A_2A_ receptor agonism or Ba^2+^ oppose this ‘protective’ mechanism in the injured brain slice to control neuronal over‐excitation during energy failure, thus enhancing glutamate release probability during the first minutes of OGD as compared with control slices.

Concerning the nature of the inward current/s underlying the AD, data in the literature demonstrated that I_h_ increase during prolonged OGD is mostly due to AMPA receptor plus NMDA receptor activation (Rossi et al., 2000), with a contribution of GABA_A_ receptor‐mediated Cl^−^ currents, depending on the experimental conditions used (Allen et al., 2004). As we used a low Cl^−^‐based pipette solution, GABA_A_ receptor activation in our recording conditions would result in outward currents, unlikely to contribute to overall AD current. Among the two glutamate‐dependent components of the AD signal, the NMDA receptor‐ and the AMPA receptor‐dependent, the major part is likely to be due to the NMDA receptor subtype because of: 1) the removal of Mg^2+^ block upon OGD‐induced depolarization; 2) higher affinity of NMDA receptors for glutamate; and 3) the lack of receptor desensitization upon prolonged agonist exposure, unlike in the case of AMPA receptors. Thus, most of the AD current is likely carried by Na^+^ and Ca^2+^ ion entry which triggers a variety of deleterious events ultimately leading to neuronal death. This is in line with previous data showing a close temporal correlation between ionic changes in the ischaemic hippocampus and glutamate outflow (Rossi et al., 2000). Noticeably, inward deflections in the direct current potential, used to detect in vivo AD, go along with extracellular striatal glutamate increase in the dialysate of rats subjected to brief (3–5 min), repeated bilateral ischaemic episodes (Ueda et al., 1992).

In the present study, we also get insight into the mechanisms by which A_2A_ receptors modulate functional impairment during an ischaemic‐like insult in vitro by measuring a number of electrophysiological parameters that are affected by OGD. We simultaneously recorded I_h_, R_m_ and E_rev_ in order to detect the timeline of electrical changes induced by the insult. We found that, during OGD, R_m_ decreased and E_rev_ depolarized. In particular Rm decrease, consistent with an increase in membrane leakage_,_ preceded the I_h_ increase induced by OGD. We conclude that, in striatal MSNs, OGD‐induced energy failure and the consequent loss of ion gradients across the cell membrane leads to overall channel opening (R_m_ decrease) and [K^+^]_o_ accumulation (E_rev_ depolarization) leading to AD and irreversible neuronal damage.

In some cells (67%), AD was preceded by a spontaneous burst of APs. This phenomenon has been previously described by others and is related to OGD‐induced depolarization leading to the activation of voltage‐dependent Na^+^ channels (Guatteo et al., 1998; Jarvis et al., 2001), but the reason why some cells fire spontaneous APs and some others do not, is not clear. We hypothesize that, if membrane depolarization is slow enough (for example due to the fact that the recorded cell is near the surface of the slice so that fluid perfusion is more effective in removing excess extracellular K^+^ nearby the recorded cell) to activate Na^+^ channels before causing their inactivation, some APs may arise before the neurons become electrically unexcitable. On the other hand, if cells recorded are deeper inside the brain slice, [K^+^]_o_ may rise more quickly thus leading directly to Na^+^ channel inactivation before any significant activity starts.

We then investigated the influence of different voltage‐gated ion channels on AD appearance by using a pharmacological approach. When OGD was carried out in the presence of the Na^+^ channel blocker TTX, AD latency (the time at which I_h_ changes more than 20%) was unchanged in comparison to control OGD slices, but the time at which AD reached its maximal negative peak was significantly delayed and AD amplitude was reduced, indicating a slower AD deflection in the presence of TTX. Hence, our data demonstrate that voltage‐dependent Na^+^ channels are not involved in initial changes in membrane permeability leading to AD initiation, but, however, they accelerate and exacerbate the achievement of maximal AD amplitude.

It is noteworthy that, when OGD was carried out in the presence of extracellular Ba^2+^, ramp currents still increased in the pre‐AD phase, demonstrating that, at least in these conditions, OGD not only activates K^+^ currents but, in addition, other conductances just before the I_h_ drops. We hypothesize that a possible candidate for this increased conductance during OGD is Cl^−^. Indeed, MSNs are GABAergic and the large ramp‐current increase observed in the pre‐AD phase when in extracellular Ba^2+^ is likely due to huge GABA release during early excitotoxicity producing the activation of many GABA_A_ receptors. A significant contribution of GABA to the AD current has been demonstrated before (Allen et al., 2004).

We also measured sEPSCs before and during OGD in different experimental conditions, in order to gain insight into the presynaptic and postsynaptic effects of OGD and/or A_2A_ receptors during the pre‐AD phase. We found that OGD insults, carried out in control conditions, significantly reduced sEPSC frequency, indicating a reduction of neurotransmitter release during the first phases of OGD (Arcangeli et al., 2013). These data are consistent with previous results demonstrating that A_1_ receptor agonists inhibit glutamate release (Oliet & Poulain, 1999). Indeed, during hypoxic/ischaemic conditions, the huge release of endogenous adenosine leads to robust A_1_ receptor activation which, in turn, reduces vesicular neurotransmitter release (Pedata et al., 2016). On the other hand, when we performed OGD in the presence of the A_2A_ receptor agonist CGS21680, the reduction in sEPSC frequency observed in the first minutes of the insult was prevented. This is in line with previous data showing that A_2A_ receptor activation facilitates neurotransmitter release either during normoxic (Lopes et al., 2002) or ischaemic (Gui et al., 2009; Melani et al., 2003) conditions. In particular, the work by Gui and collaborators demonstrated that A_2A_ receptor‐KO mice presented a reduced glutamate outflow in the striatum after transient MCAo which correlates with reduced neurological deficit and cerebral infarct volume, thus suggesting that the protection by A_2A_ receptor inhibition is associated with the suppression of glutamate‐dependent toxicity. Of note, Ba^2+^ also prevented the OGD‐induced reduction in sEPSC frequency, in line with the fact that K^+^ channel block induces neuronal depolarization and thus facilitates neurotransmitter release. Finally, OGD did not altered sEPSC amplitude in any of the experimental conditions tested, indicating no changes in the expression of postsynaptic AMPA receptors or NMDA receptors.

In conclusion, we demonstrated here that A_2A_ receptor blockade during OGD protects striatal MSNs by reducing AD amplitude and delaying AD appearance possibly by enhancing K^+^ currents. Our data could help to develop A_2A_ receptor antagonists as new pharmacological tools for the treatment of acute ischaemic damage during brain ischaemia.

## CONFLICT OF INTEREST

The authors declare no conflict of interests.

## AUTHOR CONTRIBUTIONS

EC conceived the project and performed the experiments. EC and AJG analysed the data and wrote the manuscript.

## DECLARATION OF TRANSPARENCY AND SCIENTIFIC RIGOUR

This Declaration acknowledges that this paper adheres to the principles for transparent reporting and scientific rigour of preclinical research as stated in the BJP guidelines for Design & Analysis and Animal Experimentation, and as recommended by funding agencies, publishers and other organizations engaged with supporting research.

## Supporting information


**Table S1.** Table of statistical data analysis information.

**Figure S1** No significant differences were found in the temperature of the recording chamber between different experimental groups. Pooled data (median ± 95% confidence interval: CI) of the temperature recorded (in °C) in the recording chamber (calculated by averaging the values measured at the start and end of experiment) in striatal slices where OGD was carried out in control conditions (ctrl, n = 15) or in the presence of SCH58261 (SCH: 10 μM, n = 5), CGS21680 (CGS: 1 μM, n = 5), Ba^2+^ (2 mM, n = 5) or tetrodotoxin (TTX: 1 μM, n = 5). Dunn's multiple comparisons test.

## Data Availability

The data that support the findings of this study are available from the corresponding author upon reasonable request.
