# Supplementary material for: Selective block of adenosine A2A receptors prevents ischaemic‐like effects induced by oxygen and glucose deprivation in rat medium spiny neurons
Source: Br J Pharmacol. 2022 Jul 27;179(20):4844–56. doi: 10.1111/bph.15922 (PMC9796695; doi:10.1111/bph.15922)
Supplement: Supplementary file 1 — Table S1. Table of statistical data analysis information. [file BPH-179-4844-s001.docx]

| Figure number | Normality test | Passed normality test? | Test | Descriptive statistics | H value | Sample size | P value  (ANOVA) | Post hoc test | P values |
| --- | --- | --- | --- | --- | --- | --- | --- | --- | --- |
| **1D**  **upper panel (I_h_)** | Shapiro-Wilk test | No | Wilcoxon | Median ±95% CI |  | N_1,2_=15  21 cells from 21 slices from 15 animals | N/A | N/A | P_(pre-OGD vs OGD)_<0.0001 |
| **1D**  **middle panel (R_m_)** | Shapiro-Wilk test | No | Wilcoxon | Median ±95% CI |  | N_1,2_=15  21 cells from 21 slices from 15 animals | N/A | N/A | P_(pre-OGD vs OGD)_<0.0001 |
| **1D**  **lower panel (E_rev_)** | Shapiro | Yes | Wilcoxon | Median ±95% CI |  | N_1_=15  21 cells from 21 slices from 15 animals | N/A | N/A | P_(pre-OGD vs OGD)_<0.0001 |
| **1E** | Shapiro-Wilk test | No | Dunn’s multiple comparison test | Median ±95% CI |  | N_Ctrl_=15  21 cells from 21 slices from 15 animals | N/A | N/A | P _(Ih vs. Rm)_  =0.0121 |
| **2D** | Shapiro-Wilk test | no | None | Median ±95% CI |  | N_1_=11 out of 21  21 cells from 21 slices from 15 animals | N/A | N/A | N/A |
| **3A** | Shapiro | Yes | Dunn’s multiple comparison test | Median ±95% CI |  | N_Ctrl_=14  21 cells from 21 slices from 14 animals  N_SCH_=5  12 cells from 12 slices from 5 animals  N_CGS_=5  11 cells from 11 slices from 5 animals  N_Ba2+_=5  10 cells from 10 slices from 5 animals  N_TTX_=5  10 cells from 10 slices from 5 animals | N/A | N/A | P(_ctr vs SCH)_=0.0437 |
| **3B** | Shapiro-Wilk test | no | Dunn’s multiple comparison test | Median ±95% CI |  | Same as above | N/A | N/A | P(_ctr vs SCH)_=0.0378  P(_ctr vs TTX)_=0.0002  P(_SCH vs Ba_^2+^_)_=0.0305  P(_Ba_^2+^ _vs TTX)_=0.0003 |
| **3C** | Shapiro-Wilk test | no | Dunn’s multiple comparison test | Median ±95% CI |  | Same as above | N/A | Bonferroni | P(_ctr vs SCH)_=0.0432  P(_SCH vs CGS) <_0.0016  P(_ctr vs TTX) <_0.0015 |
| **3D** | Shapiro | Yes | Dunn’s multiple comparison test | Median ±95% CI |  | Same as above | N/A | N/A | P(_ctr vs SCH)_=0.0461 |
| **3E** | Shapiro | no | Dunn’s multiple comparison test | Median ±95% CI |  | Same as above | N/A | N/A | P(_ctr vs TTX)_=0.0001 |
| **4D**  **upper panel (I_h_)** | Shapiro | yes | Paired Student’s *t*-test | Mean±SEM |  | N=5  10 cells from 10 slices from 5 animals | N/A | N/A | P(_ctr vs Ba2+)_=0.0019  P(_Ba2+ vs OGD)_=0.0047 |
| **4D**  **middle panel (R_m_)** | Shapiro | yes | Paired Student’s *t*-test | Mean±SEM |  | N=5  10 cells from 10 slices from 5 animals | N/A | N/A | P(_ctr vs Ba2+)_=0.0018  P(_Ba2+ vs OGD)_=0.0061 |
| **4D**  **lower panel (E_rev_)** | Shapiro | yes | Paired Student’s *t*-test | Mean±SEM |  | N=5  10 cells from 10 slices from 5 animals | N/A | N/A | P(_ctr vs Ba2+)_=0.0011  P(_Ba2+ vs OGD)_=0.0004 |
| **5B**  **First panel (ctrl)** | Shapiro | no | Wilcoxon | Median ±95% CI |  | N_ctrl_=13  15 cells from 15 slices from 13 animals | N/A | N/A | P=0.0215 |
| **5B**  **second panel (SCH)** | Shapiro | Yes | Wilcoxon | Median ±95% CI |  | N_SCH_=5  7 cells from 7 slices from 5 animals | N/A | N/A | P=0.0313 |
| **5B**  **third panel (CGS)** | Shapiro | no | Wilcoxon | Median ±95% CI |  | N_CGS_=5  10 cells from 10 slices from 5 animals | N/A | N/A |  |
| **5B**  **fourth panel (Ba2+)** | Shapiro | Yes | Wilcoxon | Median ±95% CI |  | N_Ba2+_=5  10 cells from 10 slices from 5 animals | N/A | N/A |  |
| **5B**  **fifth panel (TTX)** | Shapiro | no | Wilcoxon | Median ±95% CI |  | N_TTX_=5  9 cells from 9 slices from 5 animals | N/A | N/A |  |
| **5C**  **First panel (ctrl)** | Shapiro | Yes | Wilcoxon | Median ±95% CI |  | N_ctrl_=13  15 cells from 15 slices from 13 animals | N/A | N/A |  |
| **5C**  **second panel (SCH)** | Shapiro | Yes | Wilcoxon | Median ±95% CI |  | N_SCH_=5  7 cells from 7 slices from 5 animals | N/A | N/A |  |
| **5C**  **third panel (CGS)** | Shapiro | Yes | Wilcoxon | Median ±95% CI |  | N_CGS_=5  10 cells from 10 slices from 5 animals | N/A | N/A |  |
| **5C**  **fourth panel (Ba2+)** | Shapiro | Yes | Wilcoxon | Median ±95% CI |  | N_Ba2+_=5  10 cells from 10 slices from 5 animals | N/A | N/A |  |
| **5C**  **fifth panel (TTX)** | Shapiro | Yes | Wilcoxon | Median ±95% CI |  | N_TTX_=5  9 cells from 9 slices from 5 animals | N/A | N/A |  |
| **suppl fig1** | Shapiro | Yes | Dunn’s multiple comparison test | Median ±95% CI |  | See Figure 3c | N/A | N/A |  |
