# Supplementary figures and images for: Selective block of adenosine A2A receptors prevents ischaemic‐like effects induced by oxygen and glucose deprivation in rat medium spiny neurons
Source: Br J Pharmacol. 2022 Jul 27;179(20):4844–56. doi: 10.1111/bph.15922 (PMC9796695; doi:10.1111/bph.15922)

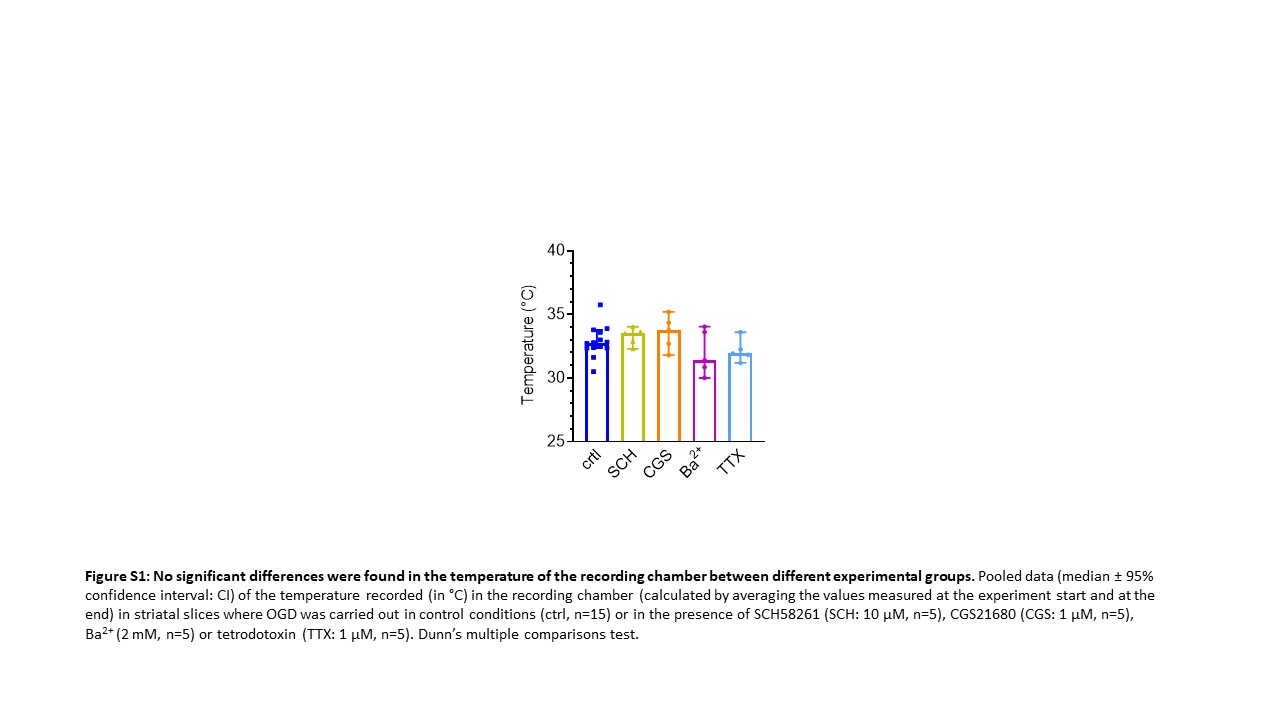

Supplement: Supplementary file 2 — Figure S1 No significant differences were found in the temperature of the recording chamber between different experimental groups. Pooled data (median ± 95% confidence interval: CI) of the temperature recorded (in °C) in the recording chamber (calculated by averaging the values measured at the start and end of experiment) in striatal slices where OGD was carried out in control conditions (ctrl, n = 15) or in the presence of SCH58261 (SCH: 10 μM, n = 5), CGS21680 (CGS: 1 μM, n = 5), Ba2+ (2 mM, n = 5) or tetrodotoxin (TTX: 1 μM, n = 5). Dunn's multiple comparisons test. [file BPH-179-4844-s002.jpg]
